# Ethyl 2-(*tert*-butoxy­carbonyl­amino)-1,3-benzothia­zole-6-carboxyl­ate

**DOI:** 10.1107/S160053681001024X

**Published:** 2010-03-24

**Authors:** Can Lei, Xin Fang, Hai-Yang Yu, Ming-Dong Huang, Jun-Dong Wang

**Affiliations:** a College of Chemistry and Chemical Engineering, Fuzhou University, Fuzhou 350108, People’s Republic of China; b Fujian Institute of Research on the Structure of Matter, State Key Laboratory of Structural Chemistry, Chinese Academy of Sciences, Fuzhou 350108, People’s Republic of China

## Abstract

In the crystal of the title compound, C_15_H_18_N_2_O_4_S, inversion dimers are formed by inter­molecular N—H⋯N hydrogen bonds and weak C—H⋯O contacts. These dimers stack up along [100] through inversion-related π–π inter­actions between thia­zole rings [centroid–centroid distance = 3.790 (2) Å] and the thia­zole and benzene rings [centroid–centroid distance = 3.845 (2) Å] and C—H⋯π contacts.

## Related literature

For benzothia­zole derivatives with anti-tumor activity, see: Brantley *et al.* (2004 ▶); Ćaleta *et al.* (2009 ▶); Mortimer *et al.* (2006 ▶) and for anti-tuberculous benzothia­zolines, see: Palmer *et al.* (1971 ▶). For related benzothia­zole structures, see: Lynch *et al.* (2002 ▶); Matković-Čalogović *et al.* (2003 ▶).
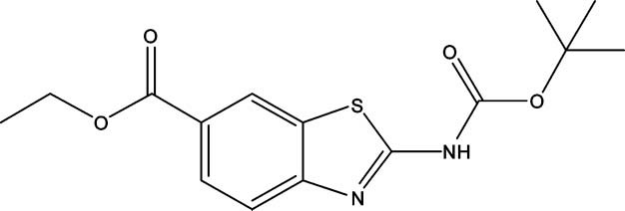


## Experimental

### 

#### Crystal data

C_15_H_18_N_2_O_4_S
*M* *_r_* = 322.37
Triclinic, 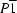

*a* = 6.3026 (13) Å
*b* = 10.791 (2) Å
*c* = 11.909 (2) Å
α = 80.58 (3)°
β = 86.61 (3)°
γ = 81.57 (3)°
*V* = 789.9 (3) Å^3^
*Z* = 2
Mo *K*α radiation
μ = 0.22 mm^−1^
*T* = 293 K
0.70 × 0.05 × 0.02 mm

#### Data collection

Rigaku Saturn 724 CCD area-detector diffractometer
Absorption correction: numerical (*NUMABS*; Higashi, 2000 ▶) *T*_min_ = 0.987, *T*_max_ = 0.995
6644 measured reflections
3469 independent reflections
2412 reflections with *I* > 2σ(*I*)
*R*_int_ = 0.049

#### Refinement

*R*[*F* ^2^ > 2σ(*F* ^2^)] = 0.088
*wR*(*F* ^2^) = 0.203
*S* = 1.11
3469 reflections
199 parameters
H-atom parameters constrained
Δρ_max_ = 0.26 e Å^−3^
Δρ_min_ = −0.30 e Å^−3^

### 

Data collection: *CrystalClear* (Rigaku, 2007 ▶); cell refinement: *CrystalClear*; data reduction: *CrystalClear*; program(s) used to solve structure: *SHELXS97* (Sheldrick, 2008 ▶); program(s) used to refine structure: *SHELXL97* (Sheldrick, 2008 ▶); molecular graphics: *ORTEX* (McArdle, 1995 ▶); software used to prepare material for publication: *SHELXL97* and *PLATON* (Spek, 2009 ▶).

## Supplementary Material

Crystal structure: contains datablocks global, I. DOI: 10.1107/S160053681001024X/si2244sup1.cif

Structure factors: contains datablocks I. DOI: 10.1107/S160053681001024X/si2244Isup2.hkl

Additional supplementary materials:  crystallographic information; 3D view; checkCIF report

## Figures and Tables

**Table 1:** Hydrogen-bond geometry (Å, °) *Cg*2 is the centroid of the C7–C12 benzene ring.

*D*—H⋯*A*	*D*—H	H⋯*A*	*D*⋯*A*	*D*—H⋯*A*
N1—H1⋯N2^i^	0.86	2.22	3.054 (14)	162
C8—H8⋯O1^i^	0.93	2.43	3.334 (14)	164
C14—H14*A*⋯*Cg*2^ii^	0.97	2.66	3.523 (18)	149

Symmetry codes: (i) 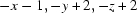; (ii) 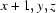.

**Fig. 1.**
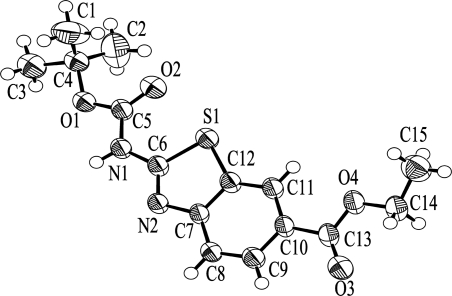
The molecular structure of the title compound. Displacement ellipsoids are shown at the 50% probability level. Hydrogen atoms are shown as spheres of arbitrary radii.

**Fig. 2.**
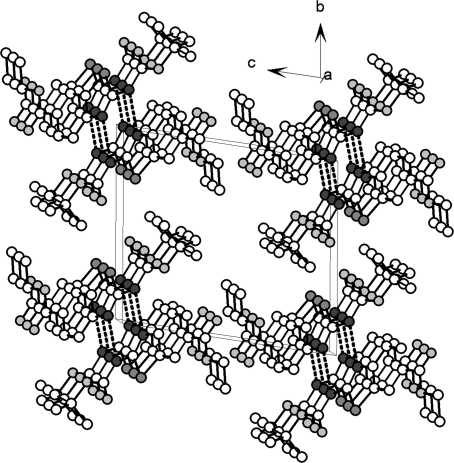
The packing diagram for the title compound. All hydrogen atoms have been omitted for clarity. Hydrogen bonds are indicated by dashed lines.

## supplementary crystallographic information

### Comment

Benzothiazole is an important moiety used in drug development because of its biological activities. A number of benzothiazole derivatives were shown to have anti-tumor (Brantley *et al.*, 2004; Mortimer *et al.*, 2006; Ćaleta *et al.*, 2009) or anti-microbial activities (Palmer *et al.*, 1971). During our development of 2-aminobenzothiazole-based Urokinase-Type Plasminogen Activator (uPA) inhibitors, we synthesized the title compound as an intermediate while its activity was not tested because it is only a fragment of our target molecule.

The molecular structure of the compound is shown in Fig. 1. The benzothiazole moiety in this structure is very similar to other benzothiazole compounds reported before (Lynch, 2002; Matković-Čalogović *et al.*, 2003). The dihedral angle between the carbonylamino group and the planar central 9-membered ring system is 7.59 (6) °, and between the central rings and the ethylcarboxylate group is 7.72 (6) °, respectively.

The packing of molecules is shown in Fig. 2. Molecules form pairs *via* N—H···N and C—H···O hydrogen bonds over crystallographic inversion symmetry. π—π stacking and C14—H14A···Cg2 hydrogen bonds (Cg2 is the benzene ring centroid) link pairs in a stacking column. In the π—π packing, Cg1···Cg1^iii^ is 3.790 (2) Å (Cg1 is the thiazole ring centroid and symmetry code iii = -x, 2-y, 2-z), the plane to plane distance of the two thiazole rings is 3.382 Å with an offset of 1.711 Å, Cg1···Cg2^iii^ is 3.845 (2) Å, the perpendicular distances of Cg1 to benzene ring is 3.369 Å, and Cg2 to thiazole ring is 3.383 Å. The hydrogen bonds are listed in Table 1, and the stacking geometries calculated with PLATON (Spek, 2009).

### Experimental

Di-tert-butyl dicarbonate (4.92 g, 22.5 mmol) and 4-dimethylamino pyridine (2.06 g, 16.9 mmol) were added to a solution of ethyl 2-aminebenzothiazole-6-carboxylate (the starting compound) (2.5 g, 11.3 mmol) in dry THF (300 ml), and stirred for 22 hours at room temperature. Then the solvent THF was evaporated, and the residue was extracted with 1 liter of dichloromethane. The dichloromethane washed with 1 N aq HCl, water, and brine, sequentially, and dried with Na_2_SO_4_. Further filtration and concentration yielded the dried compound as a yellow solid [2.61 g, yield: 72%]. The solid was dissolved in DMF and filtered. The DMF was evaporated slowly at room temperature for a week, giving colorless needle crystals.

### Refinement

All H atoms bound to C and N atoms were refined as riding, with C—H distances in the range of 0.93 to 0.97 Å and N—H distances of 0.86 Å, with *U*_iso_(H) = 1.2*U*_eq_(C, N); 1.5*U*_eq_(C_methyl_).

### Crystal data

**Table:**

C_15_H_18_N_2_O_4_S	*Z* = 2
*M**_r_* = 322.37	*F*(000) = 340
Triclinic, *P*1	*D*_x_ = 1.355 Mg m^−^^3^
Hall symbol: -P 1	Mo *K*α radiation, λ = 0.71073 Å
*a* = 6.3026 (13) Å	Cell parameters from 2354 reflections
*b* = 10.791 (2) Å	θ = 12–18°
*c* = 11.909 (2) Å	µ = 0.22 mm^−^^1^
α = 80.58 (3)°	*T* = 293 K
β = 86.61 (3)°	Needle, colorless
γ = 81.57 (3)°	0.70 × 0.05 × 0.02 mm
*V* = 789.9 (3) Å^3^	

### Data collection

**Table:**

Rigaku Saturn 724 CCD area-detector diffractometer	3469 independent reflections
Radiation source: fine-focus sealed tube	2412 reflections with *I* > 2σ(*I*)
Graphite	*R*_int_ = 0.049
Detector resolution: 28.5714 pixels mm^-1^	θ_max_ = 27.5°, θ_min_ = 3.3°
dtprofit.ref scans	*h* = −7→8
Absorption correction: numerical (*NUMABS*; Higashi, 2000)	*k* = −13→11
*T*_min_ = 0.987, *T*_max_ = 0.995	*l* = −15→15
6644 measured reflections	

### Refinement

**Table:**

Refinement on *F*^2^	Primary atom site location: structure-invariant direct methods
Least-squares matrix: full	Secondary atom site location: difference Fourier map
*R*[*F*^2^ > 2σ(*F*^2^)] = 0.088	Hydrogen site location: inferred from neighbouring sites
*wR*(*F*^2^) = 0.203	H-atom parameters constrained
*S* = 1.11	*w* = 1/[σ^2^(*F*_o_^2^) + (0.0613*P*)^2^ + 0.839*P*] where *P* = (*F*_o_^2^ + 2*F*_c_^2^)/3
3469 reflections	(Δ/σ)_max_ < 0.001
199 parameters	Δρ_max_ = 0.26 e Å^−^^3^
0 restraints	Δρ_min_ = −0.30 e Å^−^^3^

### Special details

**Table:**

Geometry. All esds (except the esd in the dihedral angle between two l.s. planes) are estimated using the full covariance matrix. The cell esds are taken into account individually in the estimation of esds in distances, angles and torsion angles; correlations between esds in cell parameters are only used when they are defined by crystal symmetry. An approximate (isotropic) treatment of cell esds is used for estimating esds involving l.s. planes.
Refinement. Refinement of *F*^2^ against ALL reflections. The weighted *R*-factor wR and goodness of fit *S* are based on *F*^2^, conventional *R*-factors *R* are based on *F*, with *F* set to zero for negative *F*^2^. The threshold expression of *F*^2^ > σ(*F*^2^) is used only for calculating *R*-factors(gt) etc. and is not relevant to the choice of reflections for refinement. *R*-factors based on *F*^2^ are statistically about twice as large as those based on *F*, and *R*- factors based on ALL data will be even larger.

### Fractional atomic coordinates and isotropic or equivalent isotropic displacement parameters (Å^2^)

**Table:**

	*x*	*y*	*z*	*U*_iso_*/*U*_eq_	
S1	−0.00627 (16)	0.75048 (10)	0.94306 (9)	0.0535 (3)	
O1	−0.5534 (4)	0.6760 (2)	1.1764 (2)	0.0535 (7)	
N1	−0.4017 (5)	0.8086 (3)	1.0445 (3)	0.0463 (7)	
H1	−0.5187	0.8593	1.0515	0.056*	
O4	0.6440 (4)	0.9028 (3)	0.6602 (2)	0.0544 (7)	
N2	−0.2502 (5)	0.9692 (3)	0.9263 (2)	0.0425 (7)	
O2	−0.2352 (5)	0.6079 (3)	1.0923 (3)	0.0736 (10)	
O3	0.5621 (5)	1.1131 (3)	0.6102 (3)	0.0702 (9)	
C1	−0.6011 (11)	0.4579 (5)	1.1729 (5)	0.101 (2)	
H1A	−0.6143	0.3772	1.2185	0.151*	
H1B	−0.4776	0.4496	1.1221	0.151*	
H1C	−0.7275	0.4866	1.1295	0.151*	
C2	−0.3890 (9)	0.5131 (6)	1.3253 (5)	0.113 (2)	
H2A	−0.4042	0.4330	1.3717	0.169*	
H2B	−0.3848	0.5759	1.3733	0.169*	
H2C	−0.2583	0.5050	1.2798	0.169*	
C3	−0.7806 (8)	0.5823 (5)	1.3176 (5)	0.0844 (17)	
H3A	−0.8094	0.5075	1.3685	0.127*	
H3B	−0.8973	0.6104	1.2671	0.127*	
H3C	−0.7654	0.6480	1.3608	0.127*	
C4	−0.5758 (7)	0.5525 (4)	1.2490 (4)	0.0599 (11)	
C5	−0.3848 (6)	0.6882 (4)	1.1045 (3)	0.0497 (9)	
C6	−0.2382 (6)	0.8519 (4)	0.9732 (3)	0.0426 (8)	
C7	−0.0652 (5)	0.9890 (3)	0.8612 (3)	0.0410 (8)	
C8	−0.0223 (6)	1.1047 (4)	0.7995 (3)	0.0483 (9)	
H8	−0.1213	1.1775	0.8004	0.058*	
C9	0.1687 (6)	1.1096 (4)	0.7373 (3)	0.0506 (9)	
H9	0.1980	1.1866	0.6962	0.061*	
C10	0.3196 (5)	1.0007 (4)	0.7350 (3)	0.0448 (9)	
C11	0.2781 (6)	0.8856 (4)	0.7956 (3)	0.0479 (9)	
H11	0.3769	0.8129	0.7938	0.057*	
C12	0.0869 (6)	0.8803 (3)	0.8590 (3)	0.0438 (8)	
C13	0.5180 (6)	1.0144 (4)	0.6628 (3)	0.0500 (9)	
C14	0.8351 (6)	0.9081 (4)	0.5871 (3)	0.0554 (10)	
H14A	0.9345	0.9534	0.6178	0.066*	
H14B	0.7982	0.9520	0.5116	0.066*	
C15	0.9356 (9)	0.7748 (5)	0.5812 (5)	0.0817 (15)	
H15A	1.0624	0.7757	0.5324	0.123*	
H15B	0.8357	0.7307	0.5512	0.123*	
H15C	0.9734	0.7326	0.6561	0.123*	

### Atomic displacement parameters (Å^2^)

**Table:**

	*U*^11^	*U*^22^	*U*^33^	*U*^12^	*U*^13^	*U*^23^
S1	0.0453 (6)	0.0497 (6)	0.0608 (6)	−0.0027 (4)	0.0103 (4)	−0.0027 (5)
O1	0.0468 (15)	0.0474 (15)	0.0610 (17)	−0.0030 (12)	0.0131 (13)	−0.0014 (13)
N1	0.0382 (16)	0.0485 (18)	0.0490 (18)	−0.0047 (13)	0.0040 (14)	−0.0009 (14)
O4	0.0382 (14)	0.0655 (18)	0.0594 (17)	−0.0122 (13)	0.0127 (12)	−0.0099 (14)
N2	0.0373 (16)	0.0482 (18)	0.0424 (16)	−0.0074 (13)	−0.0003 (13)	−0.0073 (14)
O2	0.0594 (19)	0.0573 (18)	0.090 (2)	0.0079 (15)	0.0272 (17)	0.0049 (16)
O3	0.0549 (18)	0.065 (2)	0.085 (2)	−0.0168 (15)	0.0138 (16)	0.0076 (17)
C1	0.128 (5)	0.060 (3)	0.113 (5)	−0.028 (3)	0.036 (4)	−0.013 (3)
C2	0.071 (4)	0.144 (6)	0.099 (4)	−0.015 (4)	−0.011 (3)	0.057 (4)
C3	0.072 (3)	0.070 (3)	0.097 (4)	−0.010 (2)	0.040 (3)	0.013 (3)
C4	0.054 (2)	0.049 (2)	0.071 (3)	−0.0094 (19)	0.010 (2)	0.008 (2)
C5	0.040 (2)	0.051 (2)	0.056 (2)	−0.0070 (17)	0.0093 (18)	−0.0075 (19)
C6	0.0377 (18)	0.049 (2)	0.0417 (19)	−0.0057 (15)	−0.0012 (15)	−0.0082 (16)
C7	0.0324 (17)	0.047 (2)	0.0438 (19)	−0.0050 (15)	−0.0003 (15)	−0.0094 (16)
C8	0.0395 (19)	0.046 (2)	0.059 (2)	−0.0067 (16)	0.0004 (17)	−0.0060 (18)
C9	0.045 (2)	0.052 (2)	0.055 (2)	−0.0145 (17)	0.0038 (18)	−0.0057 (19)
C10	0.0319 (18)	0.055 (2)	0.049 (2)	−0.0094 (16)	0.0022 (15)	−0.0106 (18)
C11	0.0381 (19)	0.053 (2)	0.050 (2)	−0.0017 (16)	0.0021 (16)	−0.0076 (18)
C12	0.0391 (19)	0.051 (2)	0.0417 (19)	−0.0065 (16)	0.0012 (15)	−0.0077 (16)
C13	0.0357 (19)	0.063 (3)	0.051 (2)	−0.0116 (18)	−0.0027 (16)	−0.0047 (19)
C14	0.042 (2)	0.073 (3)	0.052 (2)	−0.0156 (19)	0.0139 (18)	−0.012 (2)
C15	0.078 (3)	0.081 (3)	0.084 (4)	−0.008 (3)	0.027 (3)	−0.020 (3)

### Geometric parameters (Å, °)

**Table:**

S1—C12	1.738 (4)	C3—C4	1.509 (6)
S1—C6	1.750 (4)	C3—H3A	0.9600
O1—C5	1.332 (4)	C3—H3B	0.9600
O1—C4	1.486 (5)	C3—H3C	0.9600
N1—C5	1.369 (5)	C7—C8	1.396 (5)
N1—C6	1.382 (5)	C7—C12	1.404 (5)
N1—H1	0.8600	C8—C9	1.379 (5)
O4—C13	1.345 (5)	C8—H8	0.9300
O4—C14	1.446 (4)	C9—C10	1.402 (5)
N2—C6	1.290 (4)	C9—H9	0.9300
N2—C7	1.385 (4)	C10—C11	1.382 (5)
O2—C5	1.203 (5)	C10—C13	1.487 (5)
O3—C13	1.205 (4)	C11—C12	1.388 (5)
C1—C4	1.501 (7)	C11—H11	0.9300
C1—H1A	0.9600	C14—C15	1.495 (6)
C1—H1B	0.9600	C14—H14A	0.9700
C1—H1C	0.9600	C14—H14B	0.9700
C2—C4	1.499 (7)	C15—H15A	0.9600
C2—H2A	0.9600	C15—H15B	0.9600
C2—H2B	0.9600	C15—H15C	0.9600
C2—H2C	0.9600		
			
C12—S1—C6	87.89 (18)	N2—C6—S1	117.2 (3)
C5—O1—C4	120.7 (3)	N1—C6—S1	121.5 (3)
C5—N1—C6	122.6 (3)	N2—C7—C8	125.6 (3)
C5—N1—H1	118.7	N2—C7—C12	114.9 (3)
C6—N1—H1	118.7	C8—C7—C12	119.5 (3)
C13—O4—C14	115.5 (3)	C9—C8—C7	119.1 (4)
C6—N2—C7	110.1 (3)	C9—C8—H8	120.4
C4—C1—H1A	109.5	C7—C8—H8	120.4
C4—C1—H1B	109.5	C8—C9—C10	121.2 (4)
H1A—C1—H1B	109.5	C8—C9—H9	119.4
C4—C1—H1C	109.5	C10—C9—H9	119.4
H1A—C1—H1C	109.5	C11—C10—C9	120.1 (3)
H1B—C1—H1C	109.5	C11—C10—C13	122.5 (3)
C4—C2—H2A	109.5	C9—C10—C13	117.3 (3)
C4—C2—H2B	109.5	C10—C11—C12	119.0 (4)
H2A—C2—H2B	109.5	C10—C11—H11	120.5
C4—C2—H2C	109.5	C12—C11—H11	120.5
H2A—C2—H2C	109.5	C11—C12—C7	121.2 (3)
H2B—C2—H2C	109.5	C11—C12—S1	128.9 (3)
C4—C3—H3A	109.5	C7—C12—S1	109.9 (3)
C4—C3—H3B	109.5	O3—C13—O4	122.8 (4)
H3A—C3—H3B	109.5	O3—C13—C10	124.7 (4)
C4—C3—H3C	109.5	O4—C13—C10	112.5 (3)
H3A—C3—H3C	109.5	O4—C14—C15	107.8 (3)
H3B—C3—H3C	109.5	O4—C14—H14A	110.1
O1—C4—C2	109.9 (4)	C15—C14—H14A	110.1
O1—C4—C1	108.4 (4)	O4—C14—H14B	110.1
C2—C4—C1	113.7 (5)	C15—C14—H14B	110.1
O1—C4—C3	102.9 (3)	H14A—C14—H14B	108.5
C2—C4—C3	110.7 (5)	C14—C15—H15A	109.5
C1—C4—C3	110.7 (4)	C14—C15—H15B	109.5
O2—C5—O1	126.7 (4)	H15A—C15—H15B	109.5
O2—C5—N1	123.0 (3)	C14—C15—H15C	109.5
O1—C5—N1	110.3 (3)	H15A—C15—H15C	109.5
N2—C6—N1	121.3 (3)	H15B—C15—H15C	109.5

### Hydrogen-bond geometry (Å, °)

**Table:**

Cg2 is the centroid of the C7–C12 benzene ring.

**Table:**

*D*—H···*A*	*D*—H	H···*A*	*D*···*A*	*D*—H···*A*
N1—H1···N2^i^	0.86	2.22	3.054 (14)	162
C8—H8···O1^i^	0.93	2.43	3.334 (14)	164
C14—H14A···Cg2^ii^	0.97	2.66	3.523 (18)	149

Symmetry codes: (i) −*x*−1, −*y*+2, −*z*+2; (ii) *x*+1, *y*, *z*.
